# Revisiting definition and assessment of intestinal trans-epithelial passage

**DOI:** 10.1007/s00018-021-04000-8

**Published:** 2021-11-03

**Authors:** Hanna Ilchmann-Diounou, Marie Buleon, Valérie Bacquie, Vassilia Theodorou, Colette Denis, Sandrine Menard

**Affiliations:** 1 Toxalim (Research Centre in Food Toxicology), Université de Toulouse, INRAE, ENVT, INP-Purpan, UPS, Toulouse, France; 2 Institut National de la Santé et de la Recherche Médicale (INSERM), U1048, Institute of Cardiovascular and Metabolic Disease, Université Toulouse III Paul-Sabatier, Toulouse, France; 3 IRSD, Université de Toulouse, INSERM, INRAE, ENVT, UPS, 31024 Toulouse, France

**Keywords:** Diabetes, Exposure to luminal content, ADME, Intestinal permeability, Intestinal length, Intestinal barrier dysfunction

## Abstract

This study aims to remind that Intestinal Passage (IP) measurement is a complex task that cannot be achieved by a unique measure of an orally given exogenous marker in blood or urine. This will be illustrated in the case of NOD mice. Indeed, various methods have been proposed to measure IP. Among them ex vivo measurement in Ussing chambers of luminal to serosal fluxes of exogenous markers and in vivo measurement of exogenous markers in blood or urine after oral gavage are the more commonly used. Even though they are commonly used indifferently, they do not give the same information and can provide contradictory results. Published data showed that diabetic status in female Non Obese Diabetic (NOD) mice increased FD4 concentration in blood after gavage but did not modify FD4 fluxes in Ussing chamber. We observed the same results in our experimental conditions and tracked FD4 concentrations in blood over a kinetic study (Area Under the Curve—AUC). In vivo measurements are a dynamic process and address not only absorption (IP and intestinal surface) but also distribution, metabolism and excretion (ADME). Diabetic status in NOD mice was associated with an increase of intestinal length (absorptive surface), itself positively correlated with AUC of FD4 in blood. We concluded that increased intestinal length induced by diabetic status will extend the absorptive surface and increase FD4 concentration in plasma (in vivo measurement) despite no modification on IP of FD4 (ex vivo measurement). In addition, this study characterized intestinal function in diabetic NOD mice. Diabetic status in NOD female mice increases intestinal length and decreases paracellular IP (FSS) without affecting transcellular IP (HRP, FD4). Histological studies of small and large intestine did not show any modification of intestinal circumference nor villi and crypt size. Finally, diabetic status was not associated with intestinal inflammation (ELISA).

**Supplementary Information:**

The online version contains supplementary material available at 10.1007/s00018-021-04000-8.

## Introduction

The increasing discovery of the ubiquitous role of intestinal physiology in diverse extra-intestinal disorders raised the interest in investigating intestinal epithelial barrier functions including intestinal permeability/passage/transport/permeation, terms often used indistinctly. The definition of intestinal permeability is “the facility with which intestinal epithelium allows molecules to pass through by non-mediated passive diffusion” expressed in cm/s [2]. Intestinal passage, in a more general manner, is measured by unidirectional fluxes (expressed in mol.h^−1^.cm^−2^ or cm/s) (for review [3]). Increased intestinal passage (IP) is described in metabolic disorders like type 2 diabetes [4] but also in multiple autoimmune disorders (for review [5]). However, it is unclear whether intestinal hyper-permeability is a cause or a consequence of the disease and if it contributes to the disease onset or severity.

The fast increasing popularity of IP led to an important multiplication and diversification of methods to determine this parameter. The most popular ones relate on the detection of exogenous markers after ex vivo passage across intestinal fragment or in vivo in plasma or urine after oral gavage. Ex vivo methods necessitate devices to measure passage of exogenous markers across isolated intestinal fragments maintained in appropriate conditions of oxygenation, pH and temperature. Ussing chambers are a well-established ex vivo device to analyze unidirectional passage of exogenous markers in different tissues and different segments of intestine within a controlled surface [6]. The limitation of this technic is, first, the necessity for Ussing Chambers device, that is expensive and second, sampling of intestinal fragments or intestinal biopsies, both requiring invasive procedures.

Due to these limitations, alternative in vivo methods are used. An exogenous non-metabolized marker is given per os (oral gavage) and detected in urine or blood over a kinetic or at a fixed time point by an adapted method according to marker’s properties (radioactivity, fluorescence, etc.). Thanks to distinct chemical characteristics and degradation processes by intestinal microbiota, different markers can be used to assess absorption of different intestinal segments [2]. This method is quite affordable and easy to handle in laboratories and in clinical practices. The limitations of this technique is the absence of standardized protocols, making comparison difficult across studies, and the involvement of numerous confounding factors not always taken into account [7]. In most studies, it is assumed that the ability of the exogenous marker to pass the intestinal epithelium from the lumen to the blood or urine is the only variable in the equation. However, in vivo measurements are dynamic and reflect the presence of an exogenous marker at a specific time point or over a kinetic, in blood or urine. This dynamic process is dependent on various physiological parameters, such as Absorption (involving IP and intestinal surface), Distribution (throughout the organism), Metabolism (by microbiota and liver functions) and Excretion (kidney functions), well known in pharmacokinetics as ADME.

As previously reviewed, measuring intestinal passage is a complex task [8]. Although the exploration of intestinal passage by in vivo methods appear easily feasible and convenient, it can result in misleading conclusion if individual or group variability for confounding factors (ADME) are not properly evaluated. For this reason, in vivo measurement of intestinal passage alone is not recommended in basic animal practice when other ex vivo methods are possible [8].

The present paper aims to spread and strengthen this message by providing a concrete example of in vivo measurements that could result in misleading conclusions on intestinal passage in a mice model.

To achieve this goal, we took advantage of the Non Obese Diabetic (NOD) mice model. Indeed, published studies showed either increased IP to FITC Dextran 4KDa (FD4) (in vivo) [9] or no modification of FD4 IP (ex vivo—Ussing) [10] in diabetic female NOD mice compared to age matched non-diabetic NOD mice.

## Methods

### Mouse model

All experimental protocols were approved by local Animal Care Use Committee (Comité d’Ethique de Pharmacologie-Toxicologie de Toulouse—Midi-Pyrénées, France) registered as N°86 at the Ministry of Research and Higher Education (N° 0029/SMVT), and conducted in accordance with the European directive 2010/63/UE. Non Obese Diabetic (NOD) Shilt/J strain (Charles River, France) were housed by five per cage. Female NOD mice were chosen for the experiment since they develop more rapidly diabetes than male NOD mice (Jackson Laboratory Physiologic Data Sheet). Mice were kept at a constant temperature (22 ± 1 °C) and maintained on a 12:12 h light/dark cycle (lights on 8h00 am) on Specific and Opportunistic Pathogen Free (SOPF) conditions. Normal diet (Harlan2018, Envigo, Gannat, France) and water were available ad libitum. Blood glucose levels of NOD Shilt/J mice were monitored weekly from the tip of the tail vein with a glucose meter (AccuCheck, Roche, Mannheim, Germany). Mice with non-fasted glycemia superior to 250 mg/dL were considered as diabetic according to Jackson Laboratory Physiologic Data Sheet. After one to four weeks posterior to diabetes diagnosis, diabetic mice and non-diabetic mice from same cage and having the exact same age were attributed either to in vivo or ex vivo assessment of IP by exogenous markers. Three independent batches of animals were analyzed.

### Ex vivo passage of exogenous markers across intestinal fragments—Ussing chambers

Intestinal passage of exogenous markers across small intestine and colon was assessed as previously described [11]. Briefly, jejunal (as representative for small intestine: SI) and colonic fragments were mounted in Ussing chambers (Physiologic Instruments, San Diego, CA, USA). Tissues were bathed 2 h with oxygenated thermostated Krebs solution (Sigma, Saint Quentin Fallavier, France). Horse Radish Peroxidase 400 µg/mL (HRP 44 kDa; Sigma) and Fluorescein Sodium Salt 40 µg/mL (FSS 376 Da; Sigma) or FITC-Dextran 4 kDa 400 µg/mL (FD4 4 kDa, TdB, Sweden) were added to mucosal compartment. FSS and HRP were used as respective markers for para- and transcellular intestinal pathway based on their molecular weight [3].

Intestinal passage of total (intact and degraded) HRP was determined by ELISA. Briefly, 96-wells black plates (Greiner, Les Ulis, France) were coated with 10 µg/mL mouse polyclonal anti HRP (Abcam, Paris, France), blocked with PBS-1% bovine serum albumin (BSA), incubated with serosal compartments of Ussing chambers, detected with 10 µg/mL Rabbit polyclonal anti HRP biotin (Abcam) and revealed with SPRD-conjugated streptavidin (BD, Paris, France). Fluorescence intensity (FI) was measured by excitation: 565 nm/ emission: 666 nm using an automatic Infinite M200 microplate reader (Tecan, Männedorf, Switzerland). Intestinal passage of intact HRP was determined in 96-wells black plates, adding TMB substrate to serosal compartments of Ussing chambers, and measuring the slope within 90 s at 37 °C at 450 nm in automatic Infinite M200 microplate reader. Intestinal passage of FSS and FD4 were determined by measuring FI 485 nm/525 nm using an automatic Infinite M200 microplate reader. Results of Ussing chambers measurements are presented as permeability, expressed as cm/s.

### In vivo detection of the commonly used exogenous marker FITC-Dextran 4 kDa (FD4) in blood or plasma after oral gavage

Mice were fasted for 3 h prior to intragastric gavage with FD4 (750 µg/g of body weight in tap water). Drop of blood samples was obtained at the tip of the tail vein via microheamatrocrit capillary tubes (Hirschmann, Eberstadt, Germany) before (blank) and 15 min, 30 min, 60 min, 2 h, 3 h, 4 h after gavage. At the end of the procedure, mice were euthanized by cervical dislocation and blood was harvested in heart for plasma collection. Blood samples were diluted 1/20 and plasma 1/10 in NaCl 0.9%, and Fluorescent Intensity (FI) measured 485 nm/525 nm using an automatic Infinite M200 microplate reader. FI in blood was corrected by FI measured before gavage (blank).

### FD4 excretion in urine

For analysis of FD4 excretion in urine, mice were anesthetized by a mixture of ketamine 100 mg/kg of body weight and xylazine 10 mg/kg of body weight and maintained anesthetized by jugular catheter perfusion with 20 mg/kg/h ketamine and a flow of 0.1 mL/h. 10 mg FD4 was injected in jugular vein (100 mg/mL), arterial pressure was monitored in femoral artery. Urine was collected through a catheter inserted in bladder at 1 h and 2 h after FD4 injection. FD4 concentration was measured by FI. After 2 h mice were euthanized by cervical dislocation, blood was taken by cardiac puncture prior measurements of FI in plasma.

### Histological analysis of intestinal morphology

Intestinal fragments of duodenum, jejunum, ileum and colon were fixed in 4% formalin and included in paraffin. Paraffin sections were stained with Hematoxylin and Eosin (Eosine aqueuse 1% Labo modern, France). Tissue were screened for morphological abnormalities. Intestinal circumference was measured (Nis Element-Ar, Nikon System-Elements-Advanced research, microscope Nikon Eclipse 90 i, France).

### Fecal excretion

In the morning, mice were isolated in clean cages without bedding material but tissue for 45 min without food. Afterwards feces were numbered.

### Cytokine measurements

Cytokines were measured in jejunal (representative of small intestine: SI) or colonic fragments suspended in RIPA buffer (0.5% deoxycholate, 0.1% SDS and 1% Igepal in TBS) containing complete anti-protease cocktail (Sigma). Jejunal and colonic protein concentrations were measured using BCA uptima kit (Interchim, Montlucon, France). TNFα, Lipocalin-2, IL-10, IL-17 and IL-22 in lysate of jejunal or colonic fragments were assayed using commercial ELISA kits (R&D Systems, Lille, France) and expressed as pg/mg of protein.

### Statistical analysis

Statistical analysis were performed using GraphPad Prism version 6.04 (GraphPad Software, La Jolla, CA, USA). Results for single comparisons were displayed as box plots [min to max] and analyzed using Student’s paired *t*-test (two-tailed) or Mann–Whitney test (two-tailed) after prior Shapiro–Wilk Normality test and *F*-Test to compare variances. Multiple measurements in time or in different tissues were displayed either as kinetics with SEM or box plots [min to max] compared per family by Holm-Sidak post-test after a significant two-way ANOVA. Differences were considered significant for *p* < 0.05. Results in text were described as NOD diabetic mean ± SEM vs. NOD non-diabetic mean ± SEM for normally distributed samples and as median, [25%-quartile; 75%-quartile] in other case.

## Results

### Ex vivo intestinal passage (IP) of Fluorescein Sodium Salt (FSS) is decreased in diabetic NOD mice without modification of IP of FD4 nor Horse Radish Peroxidase (HRP)

**Fig. 1:**
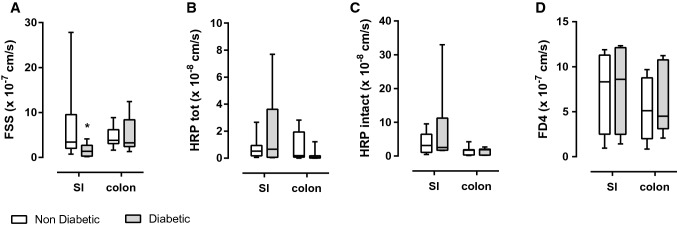
Measurements of intestinal passage (IP) in Ussing chambers in jejunum (small intestine: SI) and colonic tissue. **A** Paracellular IP (cm/s) to Fluorescein Sodium Salt (FSS), 376 Da, 40 µg/mL in serosal compartment, two-way Anova, Sidaks multiple comparison post-test, *p < 0.05, *n* = 8–12. **B** Transcellular IP (cm/s) to total Horse Radish Peroxidase (HRP), 44 kDa, 400 µg/mL in serosal compartment, *n* = 8–13. **C** Transcellular IP (cm/s) to intact Horse Radish Peroxidase (HRP), 44 kDa, 400 µg/mL in serosal compartment, *n* = 6–8. **D** IP (cm/s) to FITC-Dextran 4 kDa (FD4), 400 µg/mL in serosal compartment, *n* = 4–5

Based on their molecular weights, Fluorescein Sodium Salt (FSS-376 Da) and Horse Radish Peroxidase (HRP-44 kDa) were used as specific para and transcellular pathway markers respectively. Paracellular passage of FSS was significantly decreased in small intestine (jejunum) of diabetic mice compared to non-diabetic (ND) mice (1.36 cm/s [0293; 2718] vs. 3,420 cm/s [2045; 9525], *p* < 0.05 in small intestine and 3270 cm/s [2.42; 8405] vs. 3810 cm/s [3105; 6170] in colon, Fig. 1A). Transcellular passage of total HRP was not modified, neither in small intestine nor in colonic tissue (Fig. 1B). Intact HRP as a measure of non-lysosomal transcellular pathway was not affected either (Fig. 1C). Finally, intestinal passage of FITC-Dextran 4 kDa (FD4) (a marker commonly used in vivo) assessed in Ussing chambers was not affected by diabetic status, neither in small intestine (SI) (jejunum) nor in colon (Fig. 1D). FD4 can be considered as a marker of transcellular permeability in this model due to its molecular weight (4 kDa) and as its measured passage is not influenced by diabetic status of NOD mice like HRP and not FSS.

### FD4 concentration in plasma is increased in diabetic NOD mice after oral gavage

**Fig. 2:**
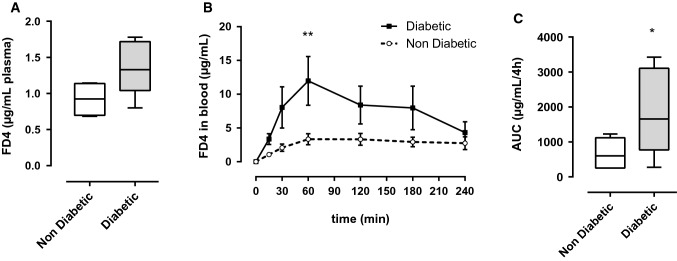
In vivo measurements of intestinal passage to FITC-Dextran 4 kDa (FD4). **A** Plasmatic FD4 concentration 4 h after gavage by 750 µg FD4/g bodyweight, unpaired students *t*-test, *p* = 0.0809, *n* = 4–5. **B** Kinetic of FD4 fluorescence in blood during 4 h after gavage by 750 µg FD4/g bodyweight, repeated-measurement two-way ANOVA, **p < 0.01, *n* = 5–6. **C** Area under the curve (AUC) of FD4 in blood during 4 h after FD4 gavage, unpaired students *t*-test, *p* = 0.0499, *n* = 5–6

Consistently with literature, in our experiments 4 h after gavage, FD4 concentration in plasma had a tendency to be increased in NOD mice with overt diabetes compared to non-diabetic (ND) NOD mice (1.370 µg/mL ± 0.17 vs. 0.919 ± 0.12, *p* = 0.0809, Fig. 2A). We completed these data by following FD4 concentration in blood over a kinetic. After oral gavage, blood samples were taken at different time points during the 4 h-period and FD4 concentrations were monitored. Although IP to FD4 in Ussing chambers were similar in intestinal tissue of diabetic and non-diabetic mice, all along the 4 h in vivo experiment FD4 concentration in blood of diabetic NOD mice was increased and a significant difference is observed 1 h after gavage (11.975 µg/mL ± 3.61 vs. 3.340 ± 0.81, *p* < 0.01, Fig. 2B). The Area Under the Curve (AUC) of FD4 during 4 h was increased for diabetic NOD mice in comparison to non-diabetic mice (1882 ± 556.8 vs. 670.4 ± 167.3, *p* = 0.0499, Fig. 2C).

### Intestinal length is increased in diabetic NOD mice and correlates positively with Area Under the Curve (AUC) of FD4 concentrations in blood

To summarize, diabetic state in NOD mice does not affect FD4 passage across intestinal fragment in Ussing chamber but increases FD4 concentration in plasma after oral gavage. Those results could appear contradictory if one hypothesizes that they both address the same physiological process i.e., IP as usually assumed. However, in vivo measurements are submitted to dynamic processes and address not only Absorption (involving IP and intestinal surface) but also Distribution, Metabolism and Excretion (ADME). Once the dynamic process of this in vivo method is reminded, it clearly appears that based on plasma detection after oral gavage only, we cannot conclude on IP. To do so, other physiological parameters and processes (intestinal surface and DME) need to be addressed.

**Fig. 3:**
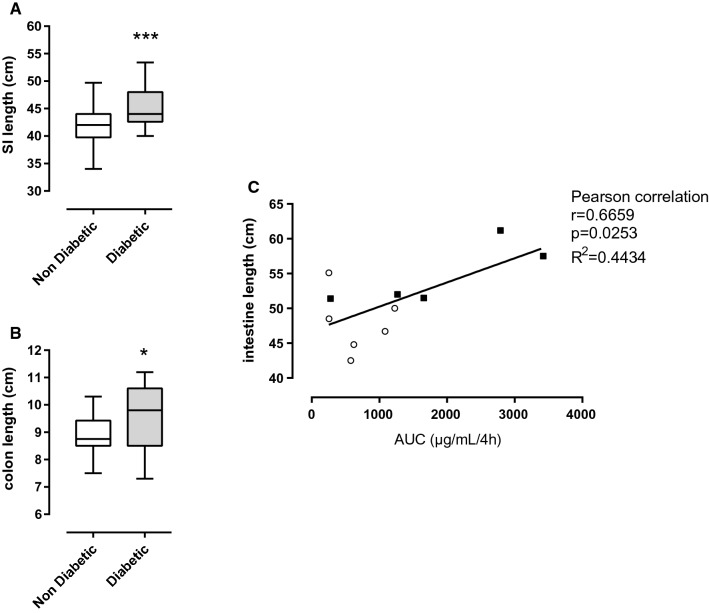
Macroscopical analysis of intestine and correlation between intestinal length and Area Under the Curve (AUC) of FITC-Dextran 4 kDa (FD4) concentration in blood, in diabetic vs. non-diabetic NOD mice. **A** Small intestine length (cm), unpaired students t-test, *p* = 0.0009, *n* = 25–26. **B** Colon length (cm), Mann–Whitney test, *p* = 0.0306, *n* = 25–26. **(C)** Total intestinal length (cm) is positively correlated with the area under the curve (AUC) of FD4 concentrations during the 4 h after FD4 gavage, Pearson correlation, *r* = 0.6659, *R*^2^ = 0.4434, *p* = 0.0253, *n* = 11, non diabetic: empty circles, diabetic: filled squares

The absorptive surface is an important confounding factor of Absorption. So, we wondered if the intestinal morphology was modified by diabetes in NOD mice compared to non-diabetic NOD mice. Surprisingly small intestine (45.24 cm ± 0.72 vs. 41.53 ± 0.76, *p* = 0.0009, Fig. 3A) and colon (9.80 cm [8.50; 10.60] vs. 8.75 [8.5; 9.43], *p* = 0.0306, Fig. 3B) of diabetic NOD mice were significantly longer than intestine of non-diabetic NOD mice. Interestingly, total intestinal length was positively correlated with the Area Under the Curve (AUC) of FD4 concentration in blood (Pearson correlation, *r* = 0.6659, *R*^2^ = 0.4434, *p* = 0.0253, Fig. 3C). This result is of particular interest since increased intestinal length induced by diabetic status will dramatically extend the absorption surface and as a consequence increases FD4 concentration in plasma (in vivo measurement Fig. 2) despite no modification on IP of FD4 (ex vivo Fig. 1).

To gain further information, we then addressed fecal excretion, which was similar between diabetic and non-diabetic NOD mice (Supplementary Fig. 1). We also questioned FD4 excretion via urine. Intestinal pathway was bypassed using intravenous injection of FD4. Diabetic NOD mice had elevated excreted quantity of urine after 1 and 2 h (Supplementary Fig. 2A) with similar FD4 concentrations in urine the first hour and lower FD4 concentration the second hour (Supplementary Fig. 2B). As a consequence, quantity of excreted FD4 in urine was higher in diabetic mice the first hour and similar the second hour (Supplementary Fig. 2C). The consequence of this higher urinary excretion of FD4 by diabetic mice is a tendency for a lower FD4 concentration in plasma of diabetic mice 2 h after intravenous injection (Supplementary Fig. 2D).

### Diabetes is not associated with modification of microscopic architecture nor inflammation of intestine in NOD mice

**Fig. 4:**
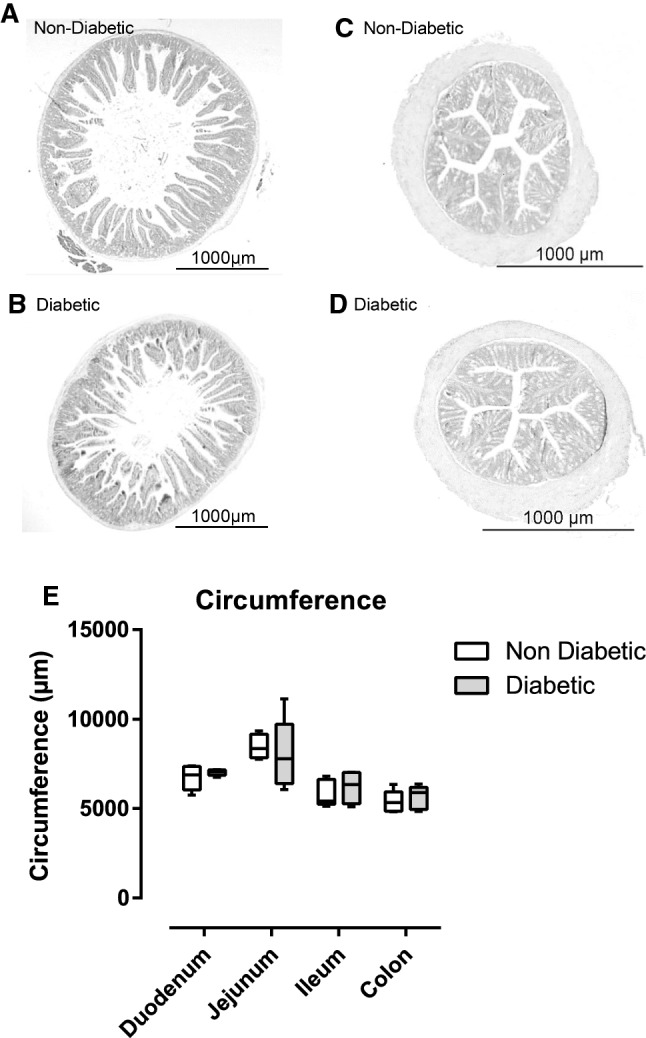
Representative cross sections of 4%-formalin, paraffin embedded haematoxylin/eosin stained jejunal (**A**, **B**) and colonic tissue (**C**, **D**), objective 2 × tissue *n* = 5. **E** Circumference of duodenum, jejunum, ileum and colon of non-diabetic and diabetic NOD mice

We also verified that morphology and histological aspect of intestine and colon were not affected by diabetic status. As microscopic architecture and inflammatory states between diabetic and non-diabetic status could equally impact ex vivo and in vivo measurement, we analyzed them to better characterize intestinal physiology of diabetic NOD mice. We did not observe any particularity or abnormality by microscopic examination of duodenal, jejunal, ileal or colonic tissues. There was no difference in circumference of small (duodenum, jejunum, and ileum) and large intestines between diabetic and non-diabetic mice excluding a major role of muscular tonus to explain our different results in in vivo and ex vivo measurements for FD4 (Fig. 4A–E).

Even though histology did not provide evidence for intestinal inflammation in diabetic NOD mice, we completed this observation by dosing cytokines (TNFα, Lipocalin-2, IL-10, IL-17 and IL-22) in lysate of colon and jejunum (small intestine: SI). As expected, cytokine levels in colonic tissues were higher. IL-10, IL-17 and IL-22 were non-detectable in lysate of SI tissue. There were no diabetes-induced modifications in cytokine concentrations (Supplementary Fig. 3).

## Discussion

This study aimed to remind that intestinal passage (IP) measurement is a complex task, which has to take into account several parameters to avoid misleading conclusions. This work is also an opportunity to provide an accurate description of intestinal functions in diabetic NOD mice.

In our opinion, the increasing popularity of the role of intestinal physiology in extra-intestinal pathologies induced some drifts in the assessment and conclusions on IP. To address and illustrate this point of view, we used exogenous markers, measured their concentrations in plasma after oral gavage (in vivo) or their passage across intestinal fragments in Ussing chamber (ex vivo). Ex vivo measurements in Ussing chambers represent an accurate method to address IP. Indeed, passage of markers across intestinal biopsies in Ussing chambers is performed within controlled surface at steady state. However, in vivo detection of markers in plasma after oral gavage is subject to a dynamic process and depend not only on Absorption (including IP and intestinal surface) but also Distribution, Metabolism, and Excretion (ADME).

We demonstrated, in Ussing chambers, that diabetic NOD mice have a decreased paracellular passage (FSS) in small intestine without modification of transcellular passage (HRP and FD4). No modification was observed in colon. This result is in accordance with a previous study demonstrating that the diabetic status in NOD mice did not affected IP of FD4 in Ussing chambers [10].

Then, we observed a tendency for increased FD4 concentrations in plasma 4 h after gavage in diabetic mice that is also consistent with literature [9]. During kinetic measurement of FD4 in blood, greatest difference in FD4 concentration was observed at 1 h post-gavage and not at 4 h. Therefore, our work highlights the utility to follow the kinetic of FD4 in blood rather than measuring FD4 concentration at a unique time point. Performing a kinetic allows to calculate Area Under the Curve (AUC) and get an overview of the response. Of note, Woting and Blaut observed that absorption kinetics are highly dependent on mouse strain [12]. At this point, our results were in accordance with previous published data showing that diabetic status in NOD mice does not affect FD4 IP but increase FD4 concentration in plasma after oral gavage.

We then investigated ADME confounding factors involved in dosage of orally given exogenous marker in plasma.

Diabetic status in NOD mice was associated with a significant increased small intestinal and colonic length that is positively correlated with FD4 AUC in blood. Interestingly, increased intestinal proliferation, intestinal weight and length have previously been described in streptozotocin- and alloxan-induced diabetes experimental models [13, 14]. The increased intestinal length in diabetic NOD mice imply greater absorptive surface that could explain elevated FD4 concentrations in blood despite no modification of FD4 IP addressed in Ussing chambers (controlled surface). The positive correlation between intestinal length and AUC of FD4 in blood of diabetic NOD mice was observed despite their higher urinary excretion at 1H after FD4 intravenous injection. Increased intestinal length has been described several years ago in different (sub-) physiological conditions. In the 70’s two studies showed that lactating rats have an increased total intestinal absorption despite decreased absorptive capacity per unit area of intestine [15, 16]. In 1975, Cripps and Williams showed that intestinal length (small and large intestine) was significantly increased in lactating rats in comparison to virgin females. Absolute absorption of leucine and glucose addressed in vivo were increased during lactation but decreased when reported to intestinal length [16]. More recently, it has been shown that cold exposure leads to major intestinal modifications as well. Indeed, small intestinal length was increased in mice housed at 6 °C in comparison to mice housed at room temperature. These modifications led to an increased absorptive surface [17]. We could not find published data on intestinal length in NOD mice. However, in a human cohort of diabetic patients, the level of glycated hemoglobin (HbA1c) was positively correlated with intestinal length [18]. This result is in accordance with our data, but question intestinal absorptive surface in T1D patients and conclusion on increasing IP in T1D patients based on in vivo measurements ([19–22] and for review [23]).

In addition to the interest of our data to revisit the definition and assessment of IP, we also provide information on intestinal functions in diabetic NOD female mice. Diabetic status in NOD female mice increased intestinal length and decreased paracellular (FSS) without affecting transcellular IP (HRP, FD4). Histological studies did not show any modification of intestinal circumference nor villi and crypt size. In our model, diabetic state was not associated with intestinal inflammation.

In summary, our work illustrates previous warning that measuring IP is not an easy task, which must take into account several parameters to avoid misleading conclusions. In vivo measurement of markers in plasma after oral gavage can only provide conclusions on IP if confounding factors (ADME) are accurately analyzed. Nevertheless, tracking of a marker in plasma after oral gavage can provide important information regarding the outcome of host physiology: absorption (intestine), distribution in organs and fluids, metabolism (liver) and excretion (kidney) and therefore the resulting systemic exposure to this marker.

## Supplementary Information

Below is the link to the electronic supplementary material.

Supplementary file1 (PDF 57 KB) Supplementary Figure 1: Fecal excretion measured over 45 minutes. Supplementary Figure 2: Urinary excretion of FITC-Dextran 4 kDa (FD4) is higher in diabetic NOD mice. FD4 (10 mg/mice) was injected in jugular vein in anesthetized mice (A) urinary volume (µL) measured 1h and 2h after intravenous injection of FD4 harvested in bladder during the experiment in anesthetized mice, n=2-3. (B) FD4 concentrations in urine (µg/mL), n=2-3. (C) Quantity of FD4 excreted as calculated from FD4 concentrations measured in urine multiplied by urine volume, n=2-3. (D) FD4 concentrations measured in plasma harvested 2h after FD4 intravenous injection, n = 2. * p<0.05, *** p< 0.001, **** p< 0.0001. Supplementary Figure 3: Cytokine concentrations in colonic and jejunum (small intestine: SI) lysate were similar between diabetic and non-diabetic NOD mice. Cytokine concentrations in pg/mg protein (A) TNFα, n=5 and (B) Lipocalin-2, n=5 in jejunum (small intestine: SI) and colon. (C) IL-10, n=5 (D) IL-17, n=5 and (E) IL-22, n=5, in colon.

## Abbreviations

ADME: Absorption Distribution metabolism and excretion
AUC: Area under the curve
FD4: FITC dextran 4KDa
FSS: Fluorescein sodium salt
HRP: Horse radish peroxidase
IP: Intestinal passage
Iv: Intravenous
NOD: Non obese diabetic
SI: Small intestine
T1D: Type 1 diabetes

## References

[1] Ilchmann H (2019) Consequences of early life adverse events on the development of non-communicable diseases in mouse models

[2] Travis S, Menzies I (2015). Intestinal permeability: functional assessment and significance. Clin Sci.

[3] Ménard S, Cerf-Bensussan N, Heyman M (2010). Multiple facets of intestinal permeability and epithelial handling of dietary antigens. Mucosal Immunol.

[4] Cani PD, Bibiloni R, Knauf C (2008). Changes in gut microbiota control metabolic endotoxemia-induced inflammation in high-fat diet-induced obesity and diabetes in mice. Diabetes.

[5] Ilchmann-Diounou H, Menard S (2020) Psychological stress, intestinal barrier dysfunctions, and autoimmune disorders: an overview. Front Immunol 11 10.3389/fimmu.2020.01823 PMC7477358 32983091

[6] Clarke LL (2009). A guide to Ussing chamber studies of mouse intestine. AJP Gastrointest Liver Physiol.

[7] Quigley EMM (2016) Leaky gut-concept or clinical entity? Curr Opin Gastroenterol. 32 10.1097/MOG.0000000000000243 26760399

[8] Galipeau HJ, Verdu EF (2016) The complex task of measuring intestinal permeability in basic and clinical science. Neurogastroenterol Motil 28 10.1111/nmo.12871 27339216

[9] Rouland M, Beaudoin L, Rouxel O (2021). Gut mucosa alterations and loss of segmented filamentous bacteria in type 1 diabetes are associated with inflammation rather than hyperglycaemia. Gut.

[10] Hadjiyanni I, Li KK, Drucker DJ (2009). Glucagon-like peptide-2 reduces intestinal permeability but does not modify the onset of type 1 diabetes in the nonobese diabetic mouse. Endocrinology.

[11] Riba A, Olier M, Lacroix-Lamandé S (2018). Early life stress in mice is a suitable model for Irritable Bowel Syndrome but does not predispose to colitis nor increase susceptibility to enteric infections. Brain Behav Immun.

[12] Woting A, Blaut M, Woting A, Blaut M (2018). Small intestinal permeability and gut-transit time determined with low and high molecular weight fluorescein isothiocyanate-dextrans in C3H mice. Nutrients.

[13] Miller DL, Hanson W, Schedl HP, Osborne JW (1977). Proliferation rate and transit time of mucosal cells in small intestine of the diabetic rat. Gastroenterology.

[14] Jervis EL, Levin RJ (1966). Anatomic adaptation of the alimentary tract of the rat to the hyperphagia of chronic alloxan-diabetes. Nature.

[15] Craft IL (1970). The influence of pregnancy and lactation on the morphology and absorptive capacity of the rat small intestine. Clin Sci.

[16] Cripps AW, Williams VJ (1975). The effect of pregnancy and lactation on food intake, gastrointestinal anatomy and the absorptive capacity of the small intestine in the albino rat. Br J Nutr.

[17] Chevalier C, Stojanović O, Colin DJ (2015). Gut microbiota orchestrates energy homeostasis during cold. Cell.

[18] Purandare A, Phalgune D, Shah S (2019). Variability of length of small intestine in Indian population and its correlation with type 2 diabetes mellitus and obesity. Obes Surg.

[19] Bosi E, Molteni L, Radaelli MG (2006). Increased intestinal permeability precedes clinical onset of type 1 diabetes. Diabetologia.

[20] Sapone A, de Magistris L, Pietzak M (2006). Zonulin upregulation is associated with increased gut permeability in subjects with type 1 diabetes and their relatives. Diabetes.

[21] Paroni R, Fermo I, Molteni L (2006). Lactulose and mannitol intestinal permeability detected by capillary electrophoresis. J Chromatogr B Analyt Technol Biomed Life Sci.

[22] Secondulfo M, Iafusco D, Carratù R (2004). Ultrastructural mucosal alterations and increased intestinal permeability in non-celiac, type I diabetic patients. Dig Liver Dis.

[23] Mønsted MØ, Falck ND, Pedersen K (2021). Intestinal permeability in type 1 diabetes: an updated comprehensive overview. J Autoimmun.

