# Supplementary figures and images for: Revisiting definition and assessment of intestinal trans-epithelial passage
Source: Cell Mol Life Sci. 2021 Nov 3;78(24):8157–64. doi: 10.1007/s00018-021-04000-8 (PMC8629865; doi:10.1007/s00018-021-04000-8)

# Supplementary Figure 1

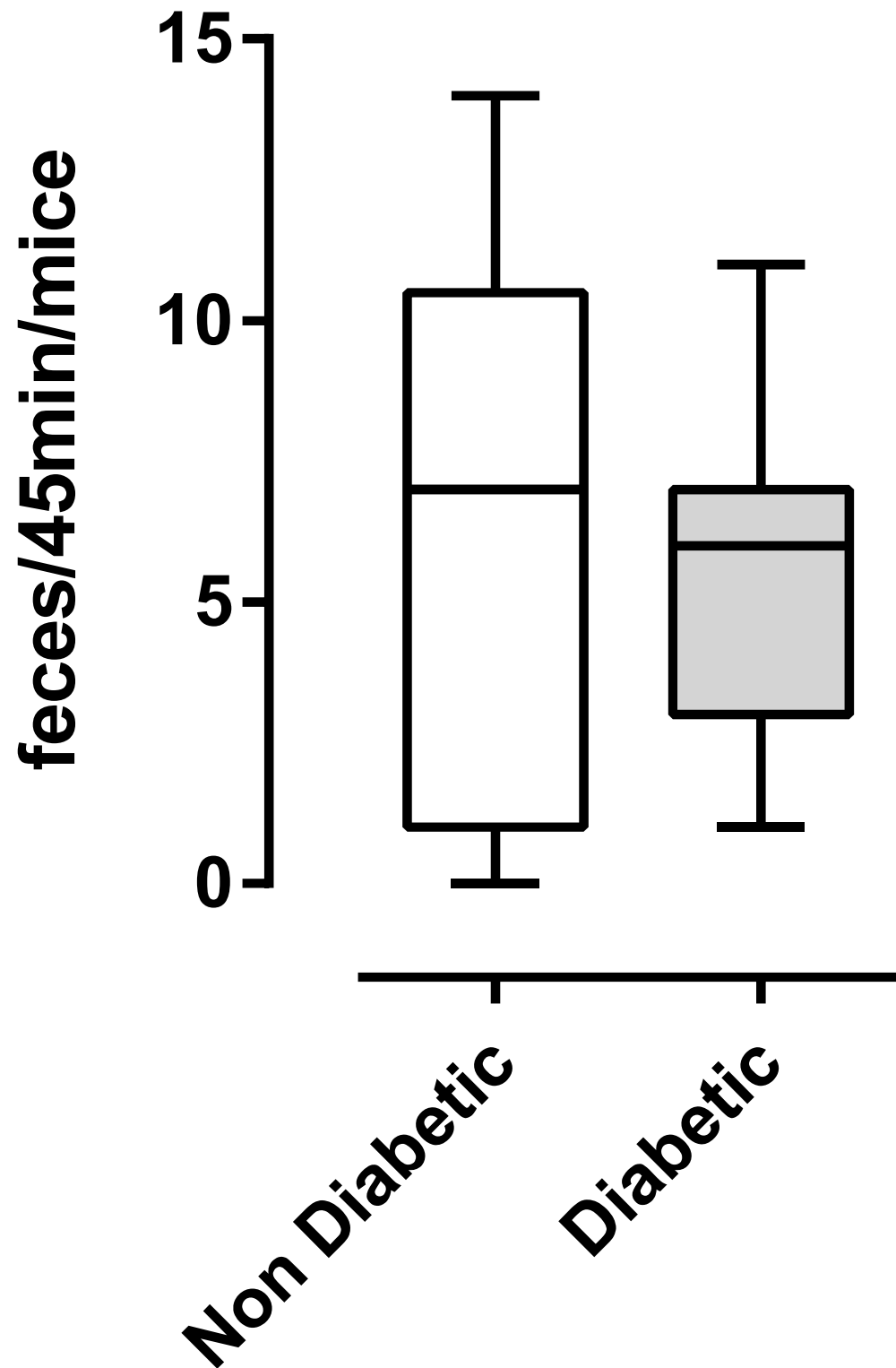

Supplementary Figure 2

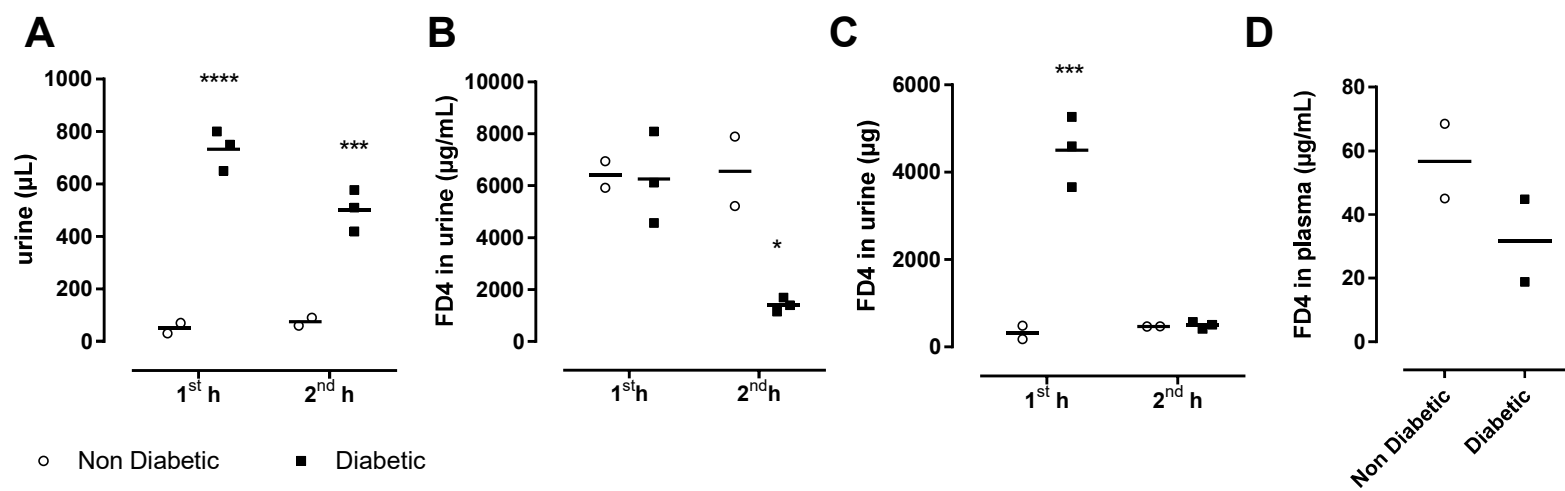

Supplementary Figure 3

**A**

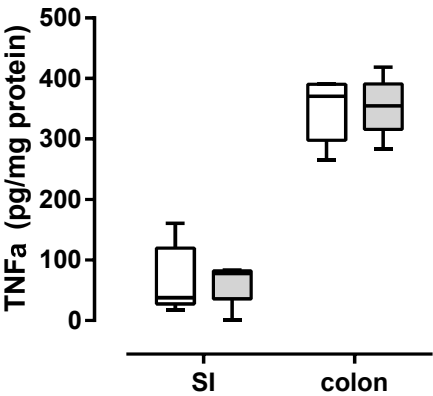

**B**

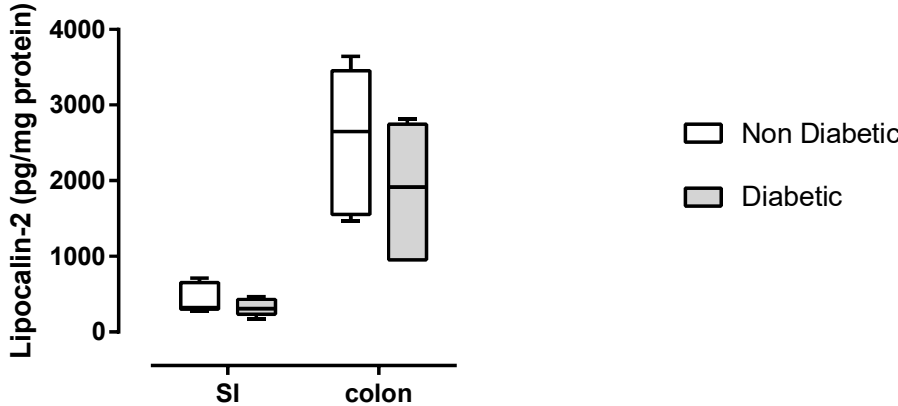

**C**

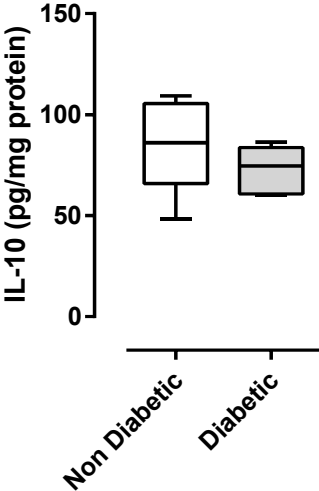

**D**

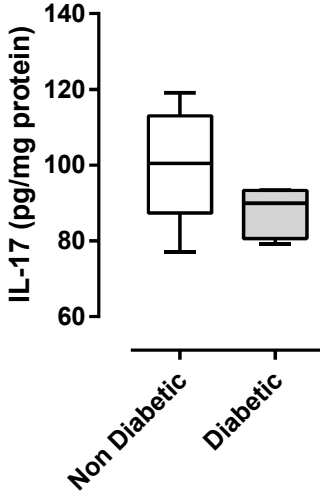

**E**

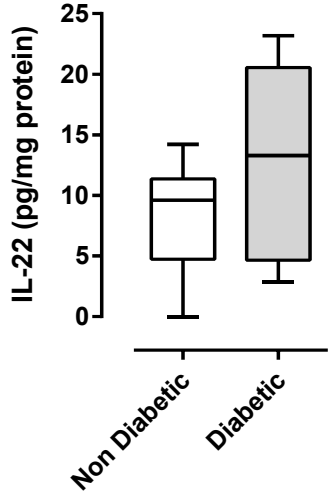

Supplement: Supplementary file 1 — Supplementary file1 (PDF 57 KB) Supplementary Figure 1: Fecal excretion measured over 45 minutes. Supplementary Figure 2: Urinary excretion of FITC-Dextran 4 kDa (FD4) is higher in diabetic NOD mice. FD4 (10 mg/mice) was injected in jugular vein in anesthetized mice (A) urinary volume (µL) measured 1h and 2h after intravenous injection of FD4 harvested in bladder during the experiment in anesthetized mice, n=2-3. (B) FD4 concentrations in urine (µg/mL), n=2-3. (C) Quantity of FD4 excreted as calculated from FD4 concentrations measured in urine multiplied by urine volume, n=2-3. (D) FD4 concentrations measured in plasma harvested 2h after FD4 intravenous injection, n = 2. * p<0.05, *** p< 0.001, **** p< 0.0001. Supplementary Figure 3: Cytokine concentrations in colonic and jejunum (small intestine: SI) lysate were similar between diabetic and non-diabetic NOD mice. Cytokine concentrations in pg/mg protein (A) TNFα, n=5 and (B) Lipocalin-2, n=5 in jejunum (small intestine: SI) and colon. (C) IL-10, n=5 (D) IL-17, n=5 and (E) IL-22, n=5, in colon. [file 18_2021_4000_MOESM1_ESM.pdf]
